# miR-195-5p Regulates Hair Follicle Inductivity of Dermal Papilla Cells by Suppressing Wnt/*β*-Catenin Activation

**DOI:** 10.1155/2018/4924356

**Published:** 2018-04-22

**Authors:** Ningxia Zhu, Keng Huang, Yang Liu, Huan Zhang, En Lin, Yang Zeng, Haihong Li, Yanming Xu, Bozhi Cai, Yanping Yuan, Yu Li, Changmin Lin

**Affiliations:** ^1^Department of Pathology and Physiopathology, Guilin Medical University, Guilin, Guangxi 541004, China; ^2^Department of Cardiology, First Affiliated Hospital, Shantou University Medical College, Shantou, Guangdong 515041, China; ^3^Department of Emergency, Second Affiliated Hospital, Shantou University Medical College, Shantou, Guangdong 515041, China; ^4^Department of Histology and Embryology, Shantou University Medical College, Shantou, Guangdong 515041, China; ^5^Department of Burn and Plastic Surgery, Second Affiliated Hospital, Shantou University Medical College, Shantou, Guangdong 515000, China; ^6^Department of Cell Biology, Shantou University Medical College, Shantou, Guangdong 515041, China; ^7^Tissue Engineering Laboratory, First Affiliated Hospital, Shantou University Medical College, Shantou, Guangdong 515041, China

## Abstract

Dermal papilla (DP) cells play a vital role in hair follicle (HF) development and postnatal hair cycling. However, the abilities are lost on further culture. Recent studies have demonstrated significant influences of posttranscriptional regulation by microRNA (miRNA) on HF development. The current study aims to investigate how miRNAs regulate Wnt/*β*-catenin to control HF inductivity of DP cells by performing microarray analysis in early- and late-passage DP cells and transfecting with miRNAs inhibitor or mimic. Results showed early-passage DP cells strongly expressed miRNAs related to inhibition of noncanonical Wnt pathways. In late-passage DP cells, miRNAs capable of inhibiting the canonical Wnt/*β*-catenin pathway were upregulated, in addition to the miRNAs targeting the noncanonical Wnt pathway. Moreover, we verified that *β*-catenin expression was downregulated by miR-195-5p overexpression in dose manner. Meanwhile LRP6 expression was downregulated in both protein and mRNA as well as the genes involved in the hair inductivity of DP cells. These results suggest that the appearance of miRNAs that suppress the Wnt/*β*-catenin pathway may be responsible for the loss of ability of DP cells in culture and miR-195-5p is the potential key factor involved in regulating HF inductivity of DP cells.

## 1. Introduction

Cultured DP cells can induce hair follicle differentiation in epithelial cells and are required for hair reconstitution to alleviate baldness [1–3]. DP cells exhibit aggregative behavior and maintaining follicle inductivity in vivo and in early passage, but further culture results in the loss of these abilities [4, 5]. Also, we have elucidated previously that early-passage cells (passage-4 cells) showed follicle-inducing ability when being injected into the back of NU/NU mice, and late-passage DP cells (passage-10 cells) failed [6]. Currently, many studies have established various approaches to maintain characteristics in vivo in cultured DP cells, including sphere formation [7, 8] or coculture with keratinocytes [9–11].

The Wnt/*β*-catenin pathway activates genes, including* CCN1*,* CCN2* [12],* OVOL1* [13], and* Versican* [14], which are essential for the maintenance of the trichogenicity of dermal cells. In DP cells, activity of the Wnt pathway is critical for DP cell-mediated hair follicle induction [15–18]: (1) many studies have explored the hair-inducing ability of DP cells under Wnt/*β*-catenin signaling activation such as a GSK-3*β* inhibitor, 6-bromoindirubin-3′-oxime (BIO), and Wnt3a [19], (2) active Wnt signaling in dermal condensates is important for hair follicle formation to proceed after induction, and inactivation of the *β*-catenin gene within the DP cells of fully developed hair follicles results in premature induction of the destructive phase of the hair cycle (catagen) and also prevents stem cells resident in the permanent portion of the follicle from regeneration of the cycling follicle [20], and (3) gene expression analysis reveals that *β*-catenin activity in the dermal papilla regulates at least two (FGF and IGF) signaling pathways that mediate the inductive effects of the DP on keratinocytes [2].

MicroRNAs (miRNAs), the small noncoding RNAs of approximately 22 nucleotides, have been implicated in regulating a number of signaling pathways, including the Wnt pathway [21]. Further evidence for the involvement of miRNAs in hair follicle development includes the differential miRNA expression between skin and follicles and the fact that mice exhibit abnormal hair follicles and cysts in skin following conditionally targeted Dicer1 gene ablation in embryonic skin progenitors [22–26]. In particular, miR-214 expression patterns have been observed in the developing epidermis, and hair follicle (HF) and keratinocyte-specific miR-214 overexpression causes a marked decrease in the number of HFs in developing skin, and a delay in anagen progression during postnatal development, together with the changes in gene expression and signaling pathway activation including Wnt, SHH, Eda, and BMP [27]. Expression of miR-31 markedly increases during anagen and decreases during catagen and telogen, suggesting miR-31 controls hair cycle-associated changes in gene expression [28]. miR-24 is expressed in HFs and has a role in hair morphogenesis by targeting Tcf-3, and miR-125b has been implicated as a repressor of bulge stem-cell differentiation by gain and loss of function in vitro by targeting Blimp1 and VDR [29].

However, the expression profile of miRNAs and potential role in regulating Wnt or related signaling pathway in different passage DP cells are unknown. Consequently, in this study we aim to investigate the expression pattern of miRNAs in early-passage and late-passage DP cell samples and their putative role with mechanism in HF inductivity.

## 2. Materials and Methods

### 2.1. Cultivation of Dermal Papilla Cells

Three human scalp specimens were obtained from one female and two male patients (aged 48, 46, and 20 years, resp.) undergoing selected face-lift surgeries. Primary culture and subculture of DP cells were performed as previously described [30]. DP cells were cultured in Dulbecco's modified Eagle's medium (DMEM) containing 10% fetal bovine serum (FBS invitrogen-Gibco) in a humidified atmosphere of 5% CO_2_ at 37°C. The fourth and tenth DP cells were chosen to represent early-passage and late-passage DP cells, and functional study of their different hair follicle inductivity abilities has been implemented in our previous work [6]. The protocols used in the study were approved by the Biomedical Ethics Committee of the First Affiliated Hospital, Shantou University Medical College, and all participants provided written informed consent.

### 2.2. RNA Extraction, Microarray Analysis, and Data Analysis

Each passage-4 and passage-10 DP cell sample derived from the above three specimens was collected from a 75 cm^2^ plastic flask after reaching confluence, at a density of 6 × 10^7^ and 6 × 10^6^ per flask, respectively. The samples were labeled as follows: DP4-a, DP4-b, DP4-c, DP10-a, DP10-b, and DP10-c. Total RNA was extracted from human DP cell samples using Trizol reagent (Invitrogen) and an miRNeasy mini kit (QIAGEN) according to manufacturer's instructions. RNA quality and quantity were measured by using a Nanodrop spectrophotometer (ND-1000, Nanodrop Technologies) and RNA integrity was determined by gel electrophoresis.

DP cell miRNA profiles were obtained by microarray analysis performed by KangChen Bio-tech and using Exiqon Array platforms. The miRCURY LNA™ microRNA Array contains 3100 capture probes, covering all human, mouse, and rat miRNAs annotated in miRBase 18.0. Scanned images were then imported into GenePix Pro 6.0 software (Axon) for grid alignment and data extraction. Expressed data were normalized using the median. After normalization, differentially expressed miRNAs were identified through volcano plot filtering. Hierarchical clustering was performed using MEV software (v4.6, TIGR). The threshold value we used to screen up- and downregulated miRNAs was a fold change ≥ 1.50 and a *P* value ≤ 0.05.

### 2.3. Q-PCR

miRNA Q-PCR was performed using a PCR master mix (Superarray) according to the manufacturer's instructions. U6 was used as the internal control. Thermal cycling conditions were as follows: 10 min at 95°C and 40 cycles of 10 s at 95°C and 60 s at 60°C. Data were analyzed by the relative quantification (2^−∆∆CT^) method. Primer sequences used for amplification are listed in Table 1.

### 2.4. miRNA Target Gene Prediction and Bioinformatics Analysis of miRNA Target Genes

TargetScan, miRBase, and miRanda miRNA target databases were used to identify putative miRNA targets. Gene ontology (GO) analysis is a functional analysis associating target genes of each microRNA. The ontology covers three domains: biological processes, cellular components, and molecular function. The GO categories are derived from Gene Ontology (http://www.geneontology.org). The pathway is based on the latest Kyoto Encyclopedia of Genes and Genomes (KEGG, http://www.genome.jp/kegg) database.

### 2.5. Cell Transfection

For transfection experiments, passage-4 DP cells were inoculated in a 6 cm dish in 1.5 mL antibiotic-free OptiMEM for 1 h. Then, cells were transfected with 0, 20, 30, 40, and 50 nM specific miRNA mimic/inhibitor (Abion), respectively, using Lipofectamine 2000 (Invitrogen, Carlsbad, CA, USA) following the manufacturer's instructions. Then 30 nM specific miRNA mimic and control oligos were transfected into the cells. All of the above-mentioned miRNA mimics/inhibitors were commercially synthesized by Ambion (USA).

### 2.6. Western Blot

Cultured cells were lysed in RIPA buffer, and lysates were analyzed using a standard western blot procedure. GAPDH was used as the loading control. PVDF membranes were incubated with anti-GAPDH (1 : 5000), anti-LRP6 (1 : 1000), anti-*β*-catenin (1 : 1000), anti-Wnt3A (1 : 1000), anti-Wnt7A (1 : 1000), anti-Noggin (1 : 1000), or anti-Wnt5A (1 : 1000) antibodies (Abcam, USA) overnight at 4°C and then washed and incubated for 1 h with secondary antibodies (Abcam, USA).

### 2.7. Statistical Analysis

The statistical significance of the differences was determined using Student's* t*-test. Data are expressed as the mean ± SD. A *P* value of <0.05 was considered statistically significant. All experiments were repeated at least three times, and for each experiment samples were analyzed in triplicate.

## 3. Experimental Results

### 3.1. Identification of Differentially Expressed miRNA in Early- and Late-Passage DP Cells

**Figure 1:**
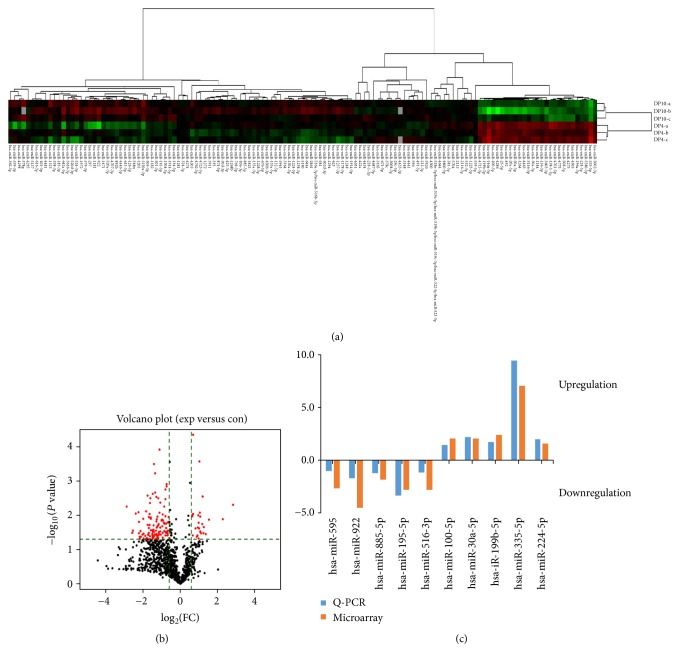
(a, b) Cluster analysis and volcano plot filtering of differentially expressed miRNAs in early versus late passage. (c) Validation of microarray data.

Our previous study demonstrated that the follicle-inducing ability of cultured DP cells declined with passage [6]. To delineate the mechanism for this decline, we profiled miRNA in early- and late-passage DP cells by microarray profiling which are deposited to the Gene Expression Omnibus (GEO) database (https://www.ncbi.nlm.nih.gov/geo/query/acc.cgi?acc=GSE77825). Of the total 1924 hsa-miRNAs (miRNAs) screened (Supplementary Table 1), we identified 27 upregulated and 106 downregulated miRNAs in 4- versus 10-passage DP cells (cutoff: *P* < 0.05; fold change > 1.5) (Table 2). Based on these differentially expressed miRNAs, a tree distinguishing DP4 cells and DP10 cells was generated by cluster analysis (Figure 1(a)). We performed volcano plot filtering for the expression in DP4 cells compared to DP10 cells and showed gene expression increased on average 1.5- to 7.0-fold and decreased 1.5- to 7.3-fold (Figure 1(b)).

### 3.2. Validation of Microarray Data by Q-PCR

In order to validate the microarray platform, we performed RT-PCR on the same RNA samples that were used for the microarrays to confirm the altered expression of ten randomly selected miRNAs that were shown by microarray analysis to be strongly up- or downregulated. Consistent with the microarray data, Q-PCR results showed miR-100-5p, miR-335-5p, miR-224-5p, miR-196b-5p, and miR-30a-5p to be upregulated and miR-516a-3p, miR-595, miR-885-5p, miR-922, and miR-195-5p to be downregulated in DP4 cells compared with DP10 cells (*P* < 0.05) (Figure 1(c)).

### 3.3. Putative Target Identification

**Figure 2:**
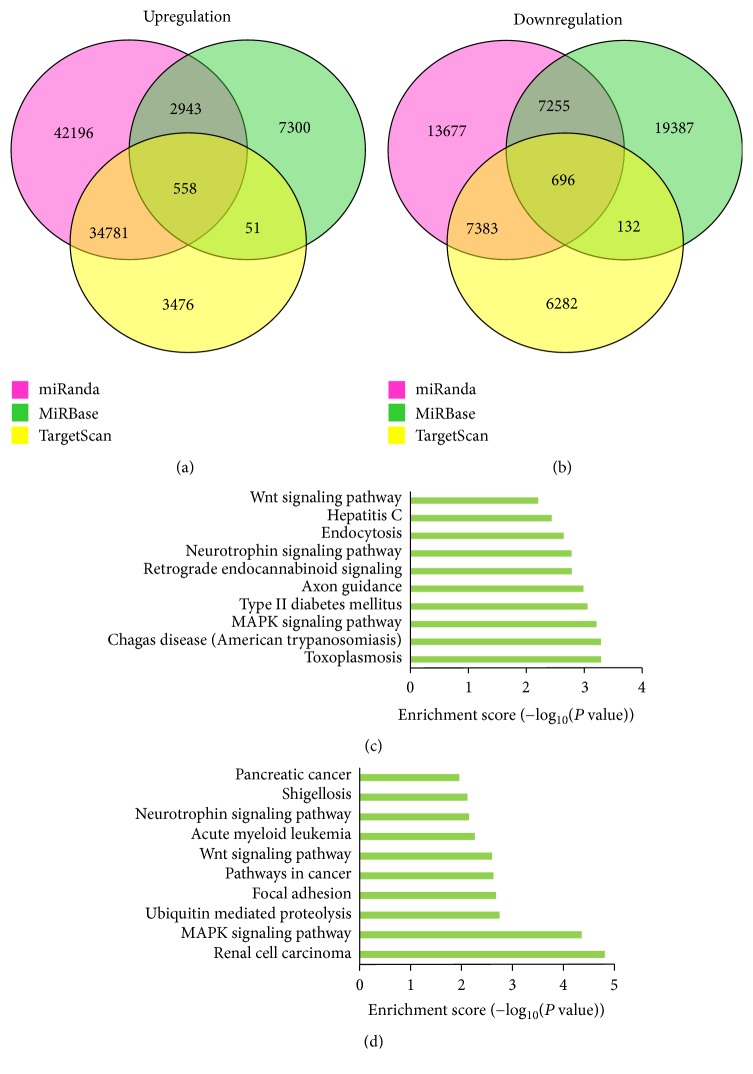
(a, b) Target genes of miRNAs by TargetScan, miRanda, and miRBase. (c, d) KEGG pathway analysis of the differentially expressed target genes.

miRNAs regulate a large number of target genes, and several databases based on various algorithms are available for predicting miRNA gene targets. We chose TargetScan, miRanda, and miRBase to predict gene targets of the 133 differentially expressed miRNAs. This resulted in the identification of 550 gene targets for upregulated miRNAs (Supplementary Table 2) and 696 gene targets for downregulated miRNAs (Figures 2(a) and 2(b), Supplementary Table 3).

### 3.4. GO and Pathway Enrichment Analysis of Differentially Expressed miRNAs

We found that the most highly enriched GOs targeted by upregulated transcripts involved signaling (ontology: biological process), intracellular components (ontology: cellular component), and protein binding (ontology: molecular function) and that the most highly enriched GOs targeted by the downregulated transcripts involved biological regulation (ontology: biological process), intracellular components (ontology: cellular component), and protein binding (ontology: molecular function) (Supplementary Table 4).

Pathway analysis indicated that 33 pathways corresponded to upregulated miRNA and 35 pathways corresponded to downregulated miRNA (Supplementary Table 5). The most enriched network was the “MAPK signaling pathway-*Homo sapiens* (human),” which corresponded to downregulated miRNAs targeting 259 genes. Notably, Wnt signaling necessary for the follicle development was included in pathways targeted by both up- and downregulated miRNA (Figures 2(c) and 2(d)).

### 3.5. miR-195-5p Regulates the Expression of *β*-Catenin

**Figure 3:**
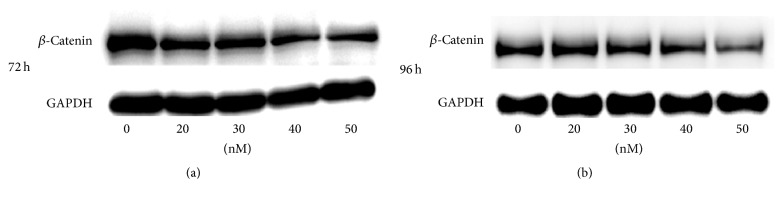
(a) *β*-Catenin expression at 72 h after transfecting miR-195-5p mimics in different dose. (b) *β*-Catenin expression at 96 h after transfecting a miR-195-5p mimics in different dose.

In the differentially expressed miRNA profile, miR-335-5p and miR-195-5p, previously reported to regulate Wnt/*β*-catenin signaling, were validated to be up- and downregulated, respectively, by Q-PCR. miR-195-5p has been shown to target Wnt3a in hepatocellular carcinoma (HCC) cells to regulate Wnt signaling and *β*-catenin transcriptional activity. Because of the Wnt signaling which was one of the key regulatory pathways for the follicle inductivity of dermal papilla, we hypothesized that the changes in miR-335-5p and miR-195-5p expression interfere with Wnt signaling. To test this hypothesis, we characterized the expression of *β*-catenin, which is upregulated in active Wnt signaling in early-passage DP cells. Due to the weak state of passage-10 DP cells, we altered the levels of miRNA only in passage-4 DP cells. Passage-4 DP cells were transfected with various concentrations of either an miR-335-5p inhibitor or an miR-195-5p mimic for 72 and 96 hours (Figures 3(a) and 3(b)). Results showed that transfection of miR-195-5p mimic did not influence cell growth and morphology in passage-4 DP cells (data not provided) and the miR-195-5p mimic treatments resulted in the downregulation of *β*-catenin expression in a dose-dependent manner (>30 nM) at 72 and 96 hours. However, transfection of DP4 cells with the miR-335-5p inhibitor did not significantly downregulate *β*-catenin expression. Therefore, the decline in ability of DP cells to induce hair follicle development could be due to the overexpression of miR-195-5p, observed in DP10 cells, resulting in the reduction of *β*-catenin in the cytoplasm and a decline in Wnt signaling.

### 3.6. miR-195-5p Targets LRP6 mRNA to Regulate LRP6 Expression in DP Cells

**Figure 4:**
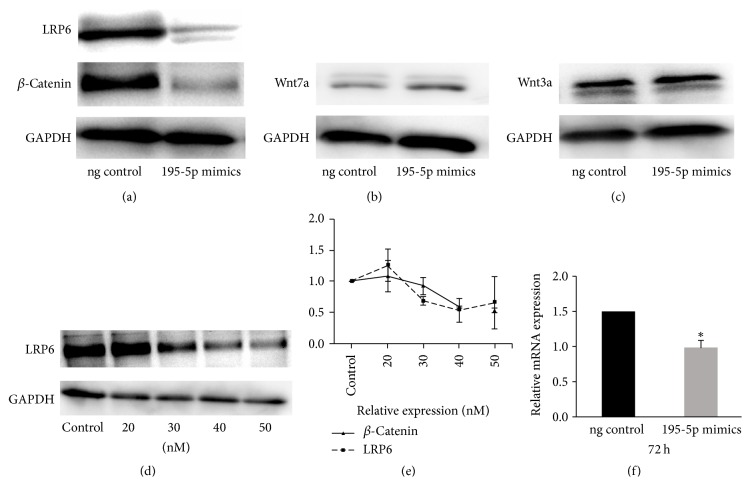
(a, b, c) The protein expression levels of LRP6, *β*-catenin, Wnt7a, and Wnt3a after being transfected with miR-195-5p mimics at 30 nM. (d, e) The expression pattern of LRP6 and *β*-catenin proteins in dose-dependent manner (>30 nM). (f) The relative expression levels of LRP6 mRNA at 72 hours by miR-195-5p mimics transfection in DP cells. ^*∗*^*P* < 0.05.

Three bioinformatic algorithms, miRanda, TargetScan, and miRBase, were used to find potential targets of miR-195-5p. Three proteins in Wnt signaling, Wnt3a, Wnt7a, and LRP6, contained potential binding sites for miR-195-5p. To test the ability of miR-195-5p to regulate Wnt3a, Wnt7a, and LRP6 expression, we transfected miR-195-5p mimics in passage-4 DP cells. Of the three proteins, only expression of LRP6 was downregulated (Figure 4(a)), while expression of Wnt3a and Wnt7a was not significantly altered (Figures 4(b) and 4(c)). To further delineate whether miR-195-5p regulates LRP6 expression, we transfected DP cells with varying doses of the miR-195-5p mimics and probed for LRP6 by western blot (Figure 4(d)). LRP6 was downregulated in dose-dependent manner (>30 nM) consistent with a decrease in *β*-catenin levels (Figure 4(e)). LRP6 mRNA levels were evaluated by Q-PCR at 24, 48, and 72 hours following transfection. Under these conditions, the relative expression levels of LRP6 mRNA were downregulated by 72 hours following miR-195-5p mimics transfection in dermal papilla cells (Figure 4(f)). Therefore, overexpression of miR-195-5p significantly decreased LRP6 expression at both the mRNA and protein levels, further indicating that LRP6 is a potential target of has-miR-195-5p.

### 3.7. miR-195-5p Inhibits the Expression of Signature Genes in DP Cells

**Figure 5:**
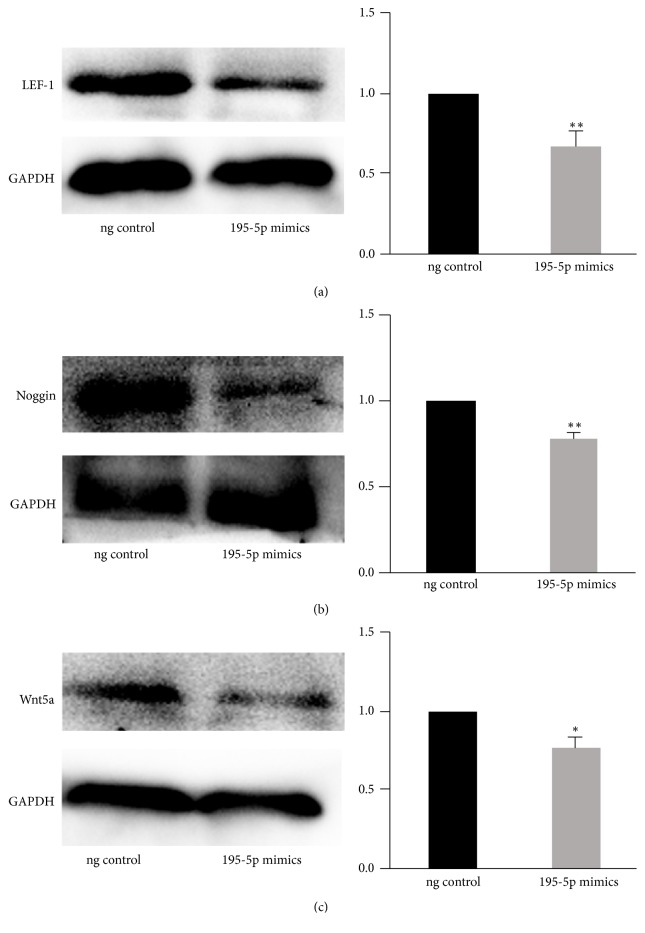
(a, b, c) The expression of LEF-1, Wnt5a, and Wnt7a after being inhibited by miR-195-5p in DP cells. ^*∗*^*P* < 0.05; ^*∗∗*^*P* < 0.01.

Next, we determined whether miR-195-5p could inhibit expression of genes downstream of Wnt signaling. The transcription factor LEF1 is a *β*-catenin binding partner in Wnt signaling, and Wnt5a and Noggin are required in DP cells for hair follicle-inductive properties. Transfection of DP cells with the miR-195-5p mimics significantly inhibited the expression of all three proteins (Figure 5), further demonstrating that miR-195-5p expression inhibits Wnt signaling.

## 4. Discussion

**Figure 6:**
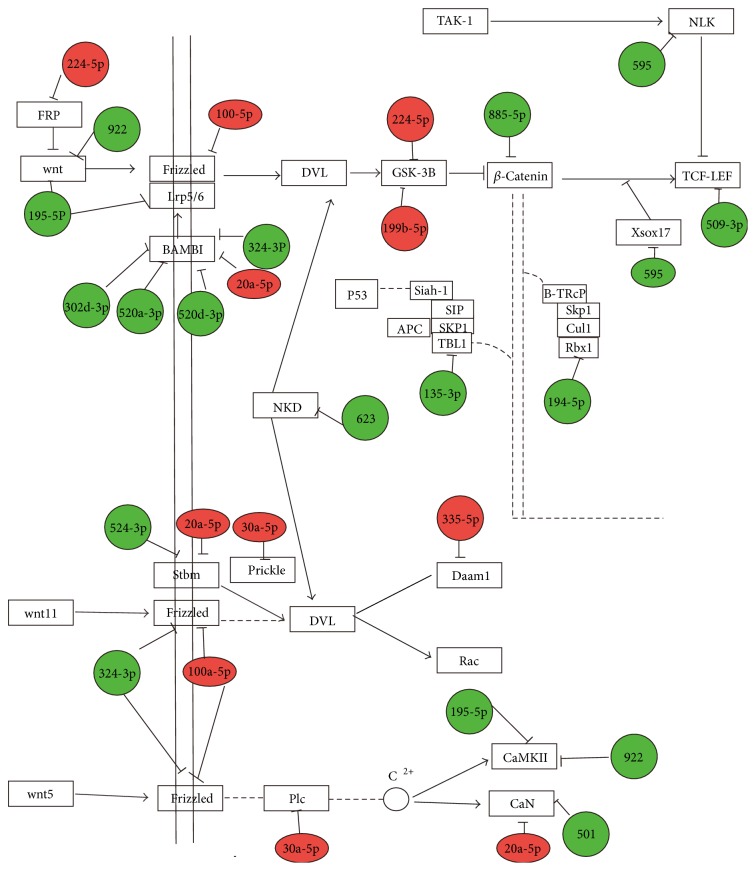
Network of differentially expressed miRNAs between the targeted genes of Wnt signaling.

Embryonic hair follicle induction and postnatal regeneration are regulated by mesenchymal-epithelial interactions involving a variety of signaling mechanisms. In this study, we characterized the relationship between miRNA and signaling proteins, such as Wnt, FGF, and BMP, based on the predicted target genes miRNAs differentially expressed between DP cells capable of (passage 4) and DP cells incapable of (passage 10) hair follicle induction. We identified 28 genes involved in Wnt signaling and 3 genes involved in FGF and BMP signaling, suggesting a large effect of miRNA on Wnt signaling. In late-passage DP cells, miRNAs that were overexpressed were predicted to inhibit canonical Wnt signaling, whereas in early-passage DP cells the miRNAs that could activate Wnt/*β*-catenin canonical signaling were downregulated and miRNAs inhibiting Wnt signaling were upregulated (Figure 6). This indicates that, with the increasing DP cell passage, miRNAs that inhibit the Wnt signaling are upregulated concomitant with the loss of DP cell hair inductivity.

The role of noncanonical Wnt signaling function in DP cells is not yet clear. Because of Wnt competition for its receptors, noncanonical Wnt signaling would inhibit Wnt/*β*-catenin canonical signaling. A recent report suggests that under normal circumstances the bone marrow microenvironment is more conducive to noncanonical Wnt signaling, compared to Wnt/*β*-catenin canonical signaling, in order to maintain the resting state of hematopoietic stem cells [31]. As the environment changes, the Wnt pathway in cells can be converted between the canonical and noncanonical pathways. Dermal papilla cells are specialized mesenchymal cells that preserve the ability of stem cells to differentiate. And Florian et al.'s study reported an unexpected shift from canonical to noncanonical Wnt signaling in mice due to elevated expression of Wnt5a in aged HSCs, which caused stem-cell aging [32]. This study shows low expression of miRNAs related to canonical Wnt pathways and high expression of miRNAs associated with inhibiting noncanonical Wnt pathways in early-passage DP cells, and in late-passage DP cells the expression levels are reversed, indicating a critical role for stem-cell-intrinsic noncanonical Wnt signaling in cells aging. Certainly, whether the expression pattern of miRNAs regulates the shift between noncanonical and canonical Wnt pathways during DP hair inductivity needs further investigation.

Many studies have shown that Wnt/*β*-catenin signaling plays a vital role in hair follicle regeneration in DP cell transplantation [33]. It has been reported that miRNAs regulate Wnt directly or indirectly by targeting genes such as Apc/Axin, Wnts, LEF1, or other signaling pathways such as wg signaling. In this study we show, using microarray and Q-PCR, that miR-195-5p expression is increased in late-passage DP cells and that overexpression decreases the expression of *β*-catenin in DP cells. Thus, miR-195-5p downregulates Wnt/*β*-catenin signaling and may be one of the reasons of late-passage DP cells losing the ability to induce hair follicles.

In this study, we predicted that Wnt3a, Wnt7a, and LRP6 were miR-195-5p target genes according to the three bioinformatic algorithms above. Of the three, only LRP6 expression is affected by miR-195-5p. This contrasts a previous study that Wnt3a is directly targeted by miR-195-5p [34]. The difference in our results could be due to the different kinds of cells. Wnt3a was predicted to be the target gene of miR-195-5p only by Target Scan, leaving out Wnt7a and LRP6. Actually, LRP6, the coreceptor of Frizzled (FZD), has been reported to be the target gene of many miRNAs, such as miR-126 and miR-183 [35, 36].

*β*-Catenin interacts with coregulators of transcription factors, including LEF-1, to activate Wnt/*β*-catenin-dependent gene transcription [37]. LEF-1 mRNA is first expressed in placode at E12.5d and upregulated in the DP. Recombination experiments using wild-type and knockout skin demonstrate a selective requirement for dermal LEF-1 expression in mediating normal hair growth [38]. In DP lacking LEF-1, primary follicles do form, but they will be arrested at an early downgrowth stage [39]. The results above indicate that overexpressed miR-195-5p in DP cells could suppress the expression of LEF-1, rendering DP cells incapable of inducing hair follicles potentially.

**Figure 7:**
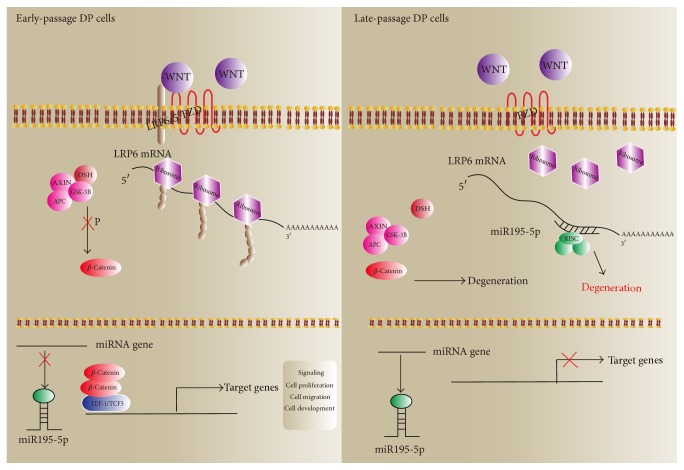
The potential mechanism of miR-195-5p regulating DP cell hair follicle inductivity.

Wnt5a and Noggin are DP cell markers for hair follicle-inductive properties [40, 41]. Wnt5a produced by dermal condensates exhibits the strongest staining as the HFs grow. Wnt5a decreases in regressing follicles, reaching its lowest level during the telogen phase [42]. Wnt5a is expressed in the developing dermal condensate of wild-type but not SHH-null embryos, indicating that Wnt5a is a target of SHH in hair follicle morphogenesis [43]. Wnt5a as an essential downstream mediator of Notch-CSL signaling is to control the hair follicle cell fate by underlying mesenchyme through a CSL-Wnt5a-FoxN1 regulatory axis [44–46]. Noggin, a BMP inhibitor, is also expressed from this compartment. A balance of these contradictory signals is thought to fine-tune the dermal messages sent to an epidermal target at this stage of development [47, 48]. Neutralization of BMPs by noggin overexpression stimulates robust formation of excess placodes, while constitutive deletion of noggin impairs the induction phase of follicle generation. In this study, we found miR-195-5p-5p inhibits the expression of LRP6, *β*-catenin, LEF-1, Wnt5a, and Noggin, which are involved in Wnt, SHH, Notch, and BMP signaling (Figure 7). Our results suggest miR-195-5p acts upstream of these signaling pathways to regulate their activation, and it is possible to regulate the follicle inductivity of DP cells.

## 5. Conclusions

The present study found expression profile of miRNAs in early- and late-passage DP cells and elucidated that upregulation of miR-195-5p could inhibit Wnt/*β*-catenin activation by targeting LRP6 and signature genes of Wnt signaling, indicating its potential involvement in HF inductivity regulation. Further functional experiments are required to understand the related mechanism.

## Figures and Tables

**Table 1:** The primer sequences used for amplification.

miRNA	Primer
U6	F: 5′GCTTCGGCAGCACATATACTAAAAT3′
	R: 5′CGCTTCACGAATTTGCGTGTCAT3′
hsa-miR-335-5p	F: 5′GGGGTCAAGAGCAATAACGAA3′
	R: 5′GTGCGTGTCGTGGAGTCG3′
hsa-miR-224-5p	F: 5′GGGGGCAAGTCACTAGTGGT3′
	R: 5′GTGCGTGTCGTGGAGTCG3′
hsa-miR-922	F: 5′GGGGCAGCAGAGAATAGGA3′
	R: 5′GTGCGTGTCGTGGAGTCG3′
hsa-miR-885-5p	F: 5′GCCCTTCCATTACACTACCCT3′
	R: 5′GTGCGTGTCGTGGAGTCG3′
hsa-miR-516a-3p	F: 5′GGGGGATGCTTCCTTTCA3′
hsa-miR-516b-3p	R: 5′GTGCGTGTCGTGGAGTCG3′
hsa-miR-595-5p	F: 5′GGGAAGTGTGCCGTGGT3′
	R: 5′CAGTGCGTGTCGTGGAGT3′
hsa-miR-195-5p	F: 5′GGGGTAGCAGCACAGAAAT3′
	R: 5′CAGTGCGTGTCGTGGAGT3′
hsa-miR-100-5p	F: 5′GCAACCCGTAGATCCGAA3′
	R: 5′CAGTGCGTGTCGTGGAGT3′
hsa-miR-30a-5p	F: 5′GGGGTGTAAACATCCTCG3′
	R: 5′CAGTGCGTGTCGTGGAG3′
hsa-miR-199b-5p	F: 5′GGGACCCCAGTGTTTAGACTAT3′
	R: 5′GTGCGTGTCGTGGAGTCG3′

**Table 2:** miRNAs that are upregulated and downregulated in 4- versus 10-passage DP cells.

Name	Fold change
hsa-miR-3150a-3p	−7.32
hsa-miR-524-3p	−5.96
hsa-miR-378g	−5.24
hsa-miR-297	−4.72
hsa-miR-922	−4.67
hsa-miR-892b	−4.65
hsa-miR-548n	−4.53
hsa-miR-1323	−4.52
hsa-miR-5190	−4.37
hsa-miR-642a-5p	−4.32
hsa-miR-520d-3p	−4.09
hsa-miR-644a	−3.95
hsa-miR-4446-5p	−3.91
hsa-miR-615-5p	−3.66
hsa-miR-2861	−3.61
hsa-miR-302d-3p	−3.59
hsa-miR-1237-3p	−3.49
hsa-miR-1181	−3.37
hsa-miR-1914-5p	−3.36
hsa-miR-379-3p	−3.34
hsa-miR-874-5p	−3.25
hsa-miR-126-5p	−3.23
hsa-miR-4707-3p	−3.20
hsa-miR-3164	−3.16
hsa-miR-1205	−3.15
hsa-miR-4298	−3.12
hsa-miR-520a-3p	−3.09
hsa-miR-194-5p	−3.07
hsa-miR-581	−3.06
hsa-miR-509-3p	−3.03
hsa-miR-1238-3p	−3.01
hsa-miR-623	−3.01
hsa-miR-338-3p	−3.00
hsa-miR-4303	−2.83
hsa-miR-516a-3p	−2.83
hsa-miR-1224-5p	−2.81
hsa-miR-616-3p	−2.81
hsa-miR-105-3p	−2.80
hsa-miR-1178-3p	−2.79
hsa-miR-195-5p	−2.79
hsa-miR-1272	−2.78
hsa-miR-1914-3p	−2.72
hsa-miR-339-3p	−2.71
hsa-miR-1471	−2.70
hsa-miR-595	−2.67
hsa-miR-4762-5p	−2.66
hsa-miR-3616-3p	−2.65
hsa-miR-132-5p	−2.65
hsa-miR-1972	−2.60
hsa-miR-501-3p	−2.57
hsa-miR-4723-5p	−2.54
hsa-miR-362-5p	−2.52
hsa-miR-185-3p	−2.48
hsa-miR-130a-5p	−2.46
hsa-miR-135a-5p	−2.44
hsa-miR-1294	−2.44
hsa-miR-676-5p	−2.42
hsa-miR-1182	−2.42
hsa-miR-1248	−2.41
hsa-miR-3945	−2.37
hsa-miR-1268b	−2.33
hsa-miR-3137	−2.29
hsa-miR-135a-3p	−2.29
hsa-miR-4481	−2.29
hsa-miR-4433-3p	−2.28
hsa-miR-4286	2.89
hsa-miR-146a-5p	4.84
hsa-miR-212-3p	−2.27
hsa-miR-3911	−2.26
hsa-miR-425-3p	−2.25
hsa-miR-769-5p	−2.24
hsa-miR-4295	−2.20
hsa-miR-1304-5p	−2.18
hsa-miR-30d-3p	−2.16
hsa-miR-4441	−2.12
hsa-miR-450b-3p	−2.11
hsa-miR-491-5p	−2.10
hsa-miR-1225-5p	−2.09
hsa-miR-4434	−2.03
hsa-miR-3926	−2.03
hsa-miR-1228-3p	−1.96
hsa-miR-1825	−1.95
hsa-miR-422a	−1.94
hsa-miR-218-1-3p	−1.93
hsa-miR-4274	−1.93
hsa-miR-324-3p	−1.90
hsa-miR-596	−1.84
hsa-miR-10b-3p	−1.83
hsa-miR-885-5p	−1.83
hsa-miR-1281	−1.76
hsa-miR-2116-3p	−1.75
hsa-miR-29c-5p	−1.74
hsa-miR-3148	−1.74
hsa-miR-378c	−1.74
hsa-miR-5008-3p	−1.74
hsa-miR-532-3p	−1.70
hsa-miR-2113	−1.70
hsa-miR-4644	−1.69
hsa-miR-4687-3p	−1.68
hsa-miR-381-5p	−1.67
hsa-miR-4300	−1.65
hsa-miR-671-5p	−1.63
hsa-miR-4651	−1.62
hsa-miR-539-5p	−1.61
hsa-miR-4800-5p	−1.60
hsa-miR-3165	−1.60
hsa-miR-518e-5p	−1.55
hsa-miR-5704	−1.51
hsa-miR-136-3p	1.55
hsa-miR-196a-3p	1.58
hsa-miR-224-5p	1.60
hsa-miR-5002-3p	1.60
hsa-miR-508-5p	1.76
hsa-miR-492	1.83
hsa-miR-1264	1.88
hsa-miR-3156-3p	1.89
hsa-miR-4275	1.95
hsa-miR-4305	1.96
hsa-miR-3605-3p	1.96
hsa-miR-20a-5p	2.00
hsa-miR-5580-5p	2.01
hsa-miR-1184	2.06
hsa-miR-100-5p	2.07
hsa-miR-30a-5p	2.09
hsa-miR-4775	2.10
hsa-miR-25-5p	2.13
hsa-miR-4701-5p	2.14
hsa-miR-4715-3p	2.25
hsa-miR-30a-3p	2.27
hsa-miR-4463	2.33
hsa-miR-199b-5p	2.39
hsa-miR-138-1-3p	2.55
hsa-miR-335-5p	7.08

## Data Availability

All sequencing and array data files are available at Gene Expression Omnibus (GEO) under the SuperSeries Accession Number GSE77825. All other data arising from this study are contained within the manuscript and supplementary information files.

## Supplementary Materials

Supplementary 1 Table S1: miRNA expression profiling data.

Supplementary 2 Table S2: upregulation expressed candidate target genes of miRNAs.

Supplementary 3 Table S3: downregulation expressed candidate target genes of miRNAs.

Supplementary 4 Table S4: GO analysis of target genes of differentially expressed miRNAs.

Supplementary 5 Table S5: KEGG analysis of target genes of differentially expressed miRNAs.

## References

[1] Jahoda C. A. B., Horne K. A., Oliver R. F. (1984). Induction of hair growth by implantation of cultured dermal papilla cells. *Nature*.

[2] Enshell-Seijffers D., Lindon C., Kashiwagi M., Morgan B. A. (2010). *β*-catenin activity in the dermal papilla regulates morphogenesis and regeneration of hair. *Developmental Cell*.

[3] Jahoda C. A. B., Reynolds A. J. (1996). Dermal-epidermal interactions: adult follicle-derived cell populations and hair growth. *Dermatologic Clinics*.

[4] Zhou L., Yang K., Xu M. (2016). Activating *β*-catenin signaling in CD133-positive dermal papilla cells increases hair inductivity. *FEBS Journal*.

[5] Yu N., Song Z., Zhang K., Yang X. (2017). MAD2B acts as a negative regulatory partner of TCF4 on proliferation in human dermal papilla cells. *Scientific Reports*.

[6] Lin C.-M., Liu Y., Huang K. (2014). Long noncoding RNA expression in dermal papilla cells contributes to hairy gene regulation. *Biochemical and Biophysical Research Communications*.

[7] Osada A., Iwabuchi T., Kishimoto J., Hamazaki T. S., Okochi H. (2007). Long-term culture of mouse vibrissal dermal papilla cells and de novo hair follicle induction. *Tissue Engineering Part A*.

[8] Zhang P., Kling R. E., Ravuri S. K. (2014). A review of adipocyte lineage cells and dermal papilla cells in hair follicle regeneration. *Journal of Tissue Engineering*.

[9] Reynolds A. J., Jahoda C. A. B. (1996). Hair matrix germinative epidermal cells confer follicle-inducing capabilities on dermal sheath and high passage papilla cells. *Development*.

[10] Inamatsu M., Matsuzaki T., Iwanari H., Yoshizato K. (1998). Establishment of rat dermal papilla cell lines that sustain the potency to induce hair follicles from afollicular skin. *Journal of Investigative Dermatology*.

[11] Kobayashi T., Fujisawa A., Amagai M., Iwasaki T., Ohyama M. (2011). Molecular biological and immunohistological characterization of canine dermal papilla cells and the evaluation of culture conditions. *Veterinary Dermatology*.

[12] Sung Y. K., Kwack M. H., Kim S. R., Kim M. K., Kim J. C. (2008). Transcriptional activation of CCN1 and CCN2, targets of canonical Wnt signal, by ascorbic acid 2-phosphate in human dermal papilla cells. *Journal of Dermatological Science*.

[13] Shin S. H., Kim D., Hwang J., Kim M. K., Kim J. C., Sung Y. K. (2014). OVO homolog-like 1, a target gene of the Wnt/*β*-catenin pathway, controls hair follicle Neogenesis. *Journal of Investigative Dermatology*.

[14] Yang Y., Li Y., Wang Y. (2012). Versican gene: Regulation by the *β*-catenin signaling pathway plays a significant role in dermal papilla cell aggregative growth. *Journal of Dermatological Science*.

[15] Andl T., Reddy S. T., Gaddapara T., Millar S. E. (2002). WNT signals are required for the initiation of hair follicle development. *Developmental Cell*.

[16] Kishimoto J., Burgeson R. E., Morgan B. A. (2000). Wnt signaling maintains the hair-inducing activity of the dermal papilla. *Genes & Development*.

[17] Leirós G. J., Ceruti J. M., Castellanos M. L., Kusinsky A. G., Balañá M. E. (2017). Androgens modify Wnt agonists/antagonists expression balance in dermal papilla cells preventing hair follicle stem cell differentiation in androgenetic alopecia. *Molecular and Cellular Endocrinology*.

[18] Kwack M. H., Ahn J. S., Jang J. H., Kim J. C., Sung Y. K., Kim M. K. (2016). SFRP2 augments Wnt/*β*-catenin signalling in cultured dermal papilla cells. *Experimental Dermatology*.

[19] Ohyama M., Kobayashi T., Sasaki T., Shimizu A., Amagai M. (2012). Restoration of the intrinsic properties of human dermal papilla in vitro. *Journal of Cell Science*.

[20] Tsai S.-Y., Sennett R., Rezza A. (2014). Wnt/*β*-catenin signaling in dermal condensates is required for hair follicle formation. *Developmental Biology*.

[21] Song J. L., Nigam P., Tektas S. S., Selva E. (2015). MicroRNA regulation of Wnt signaling pathways in development and disease. *Cellular Signalling*.

[22] Luan L., Shi J., Yu Z., Andl T. (2017). The major miR-31 target genes STK40 and LATS2 and their implications in the regulation of keratinocyte growth and hair differentiation. *Experimental Dermatology*.

[23] Miller K. J., Brown D. A., Ibrahim M. M., Ramchal T. D., Levinson H. (2015). MicroRNAs in skin tissue engineering. *Advanced Drug Delivery Reviews*.

[24] Andl T., Murchison E. P., Liu F. (2006). The miRNA-processing enzyme dicer is essential for the morphogenesis and maintenance of hair follicles. *Current Biology*.

[25] Teta M., Choi Y. S., Okegbe T. (2012). Inducible deletion of epidermal Dicer and Drosha reveals multiple functions for miRNAs in postnatal skin. *Development*.

[26] Yi R., Pasolli H. A., Landthaler M. (2009). DGCR8-dependent microRNA biogenesis is essential for skin development. *Proceedings of the National Acadamy of Sciences of the United States of America*.

[27] Ahmed M. I., Alam M., Emelianov V. U. (2014). MicroRNA-214 controls skin and hair follicle development by modulating the activity of the Wnt pathway. *The Journal of Cell Biology*.

[28] Mardaryev A. N., Ahmed M. I., Vlahov N. V. (2010). Micro-RNA-31 controls hair cycle-associated changes in gene expression programs of the skin and hair follicle. *The FASEB Journal*.

[29] Amelio I., Lena A. M., Bonanno E., Melino G., Candi E. (2013). miR-24 affects hair follicle morphogenesis targeting Tcf-3. *Cell Death and Disease*.

[30] Li Y., Li G. Q., Lin C. M., Cai X. N. (2005). One-step collagenase I treatment: an efficient way for isolation and cultivation of human scalp dermal papilla cells. *Journal of Dermatological Science*.

[31] Sugimura R., He X. C., Venkatraman A. (2012). Noncanonical Wnt signaling maintains hematopoietic stem cells in the niche. *Cell*.

[32] Florian M. C., Nattamai K. J., Dörr K. (2013). A canonical to non-canonical Wnt signalling switch in haematopoietic stem-cell ageing. *Nature*.

[33] Fuchs E. (2007). Scratching the surface of skin development. *Nature*.

[34] Chang M., Lin H., Fu H., Wang B., Han G., Fan M. (2017). MicroRNA-195-5p regulates osteogenic differentiation of periodontal ligament cells under mechanical loading. *Journal of Cellular Physiology*.

[35] Wang J., Wang X., Li Z., Liu H., Teng Y. (2014). MicroRNA-183 suppresses retinoblastoma cell growth, invasion and migration by targeting LRP6. *FEBS Journal*.

[36] Jansen F., Stumpf T., Proebsting S. (2017). Intercellular transfer of miR-126-3p by endothelial microparticles reduces vascular smooth muscle cell proliferation and limits neointima formation by inhibiting LRP6. *Journal of Molecular and Cellular Cardiology*.

[37] MacDonald B. T., Tamai K., He X. (2009). Wnt/*β*-catenin signaling: components, mechanisms, and diseases. *Developmental Cell*.

[38] Gat U., DasGupta R., Degenstein L., Fuchs E. (1998). De novo hair follicle morphogenesis and hair tumors in mice expressing a truncated *β*-catenin in skin. *Cell*.

[39] Kratochwil K., Dull M., Fariñas I., Galceran J., Grosschedl R. (1996). Lef1 expression is activated by BMP-4 and regulates inductive tissue interactions in tooth and hair development. *Genes & Development*.

[40] Rendl M., Polak L., Fuchs E. (2008). BMP signaling in dermal papilla cells is required for their hair follicle-inductive properties. *Genes & Development*.

[41] Genander M., Cook P. J., Ramsköld D. (2014). BMP signaling and its pSMADS1/5 target genes differentially regulate hair follicle stem cell lineages. *Cell Stem Cell*.

[42] Xing Y.-Z., Wang R.-M., Yang K. (2013). Adenovirus-mediated Wnt5a expression inhibits the telogen-to-anagen transition of hair follicles in mice. *International Journal of Medical Sciences*.

[43] Reddy S., Andl T., Bagasra A. (2001). Characterization of Wnt gene expression in developing and postnatal hair follicles and identification of Wnt5a as a target of Sonic hedgehog in hair follicle morphogenesis. *Mechanisms of Development*.

[44] Botchkarev V. A., Botchkareva N. V., Sharov A. A., Gilchrest B. A., Funa K., Huber O. (2002). Modulation of BMP signaling by noggin is required for induction of the secondary (Nontylotrich) hair follicles. *Journal of Investigative Dermatology*.

[45] Ji X.-Y., Wang J.-X., Liu B. (2016). Comparative transcriptome analysis reveals that a ubiquitin-mediated proteolysis pathway is important for primary and secondary hair follicle development in cashmere goats. *PLoS ONE*.

[46] Hu B., Lefort K., Qiu W. (2010). Control of hair follicle cell fate by underlying mesenchyme through a CSL-Wnt5a-FoxN1 regulatory axis. *Genes & Development*.

[47] Hu B., Lefort K., Qiu W. (1999). Noggin is a mesenchymally derived stimulator of hair-follicle induction. *Nature Cell Biology*.

[48] Woo W.-M., Zhen H. H., Oro A. E. (2012). Shh maintains dermal papilla identity and hair morphogenesis via a Noggin-Shh regulatory loop. *Genes & Development*.

